# Consequences of developmental and growth-rate plasticity within and across life stages in wood frogs (*Rana sylvatica*)

**DOI:** 10.1098/rsos.250202

**Published:** 2025-05-07

**Authors:** Sarah McKay Strobel, Eva K. Fischer, Molly C. Womack

**Affiliations:** ^1^ Department of Biology, Utah State University, Logan, UT, USA; ^2^ Department of Ecology and Evolutionary Biology, Cornell University, Ithaca, NY, USA; ^3^ Department of Neurobiology, Physiology, and Behavior, University of California Davis, Davis, CA, USA

**Keywords:** anuran, carry-over effect, crowding, metamorphosis, complex life cycle, trade-off

## Abstract

Increased trait responsiveness to the environment can provide short-term benefits but may induce delayed costs. Anurans (frogs and toads) provide an excellent system to examine phenotypic plasticity and developmental carry-over effects given their ecologically distinct life stages, which have distinct development and growth opportunities. Previous research has predominantly assessed phenotype at metamorphosis rather than within and across life stages. To address this knowledge gap, we reared wood frogs (*Rana sylvatica*) at two densities and assessed morphology and survival at multiple larval and post-metamorphic timepoints. As expected, the high-density rearing environment depressed early larval size and survivorship and delayed metamorphosis. However, compensatory growth-rate plasticity enabled high-density tadpoles to metamorphose at a similar size as low-density tadpoles. Regardless of rearing density, larval duration was negatively correlated with metamorphic mass for the earliest developers and influenced post-metamorphic survivorship and morphology, but we found evidence for a trade-off between compensatory growth and later-life survival. Our results reinforce the need to sample at multiple timepoints and life stages to understand interactions between phenotype and developmental environment. More broadly, this study contributes to understanding trade-offs and compensation associated with phenotypic plasticity, which will become even more critical given accelerating rates of global environmental change.

## 1. Background

Phenotypes are products of interactions between the genome and the environment, and variation in the strength and direction of these interactions can influence the phenotypic variation on which selection acts [1–3]. As the earliest opportunity for the genotype to interact directly with the environment, an organism’s developmental period can range from highly sensitive to insensitive to environmental conditions [4]. Phenotypic sensitivity in response to environmental cues during development is complex, and its adaptive value depends on spatial and temporal variation in the environment, reliability of cues in predicting future environments and the capacity to compensate for and/or reverse developmental responses in later life [5,6]. Developing individuals must balance the benefits of increased trait responsiveness in the current environment with the potential for direct, immediate costs associated with responsiveness, as well as with the possibility of a mismatch with the trait optimum in the later-life environment. Given the accelerating rate of global environmental change, understanding the capacity for plasticity and the consequences of shifted trait means on fitness outcomes across multiple life-history milestones, including critical developmental periods, is crucial for predicting population and species persistence.

Complex life cycles are a life-history strategy theorized to mitigate phenotypic mismatches. Predominant across multicellular organisms, complex life cycles comprise an ontogenetic morphological transformation (i.e. metamorphosis) that can serve as a decoupling mechanism that allows individuals to respond appropriately to each life stage’s unique selective pressures [7]. Anurans (i.e. frogs and toads) remain active throughout their ontogenetic shift from herbivorous, aquatic, tailed tadpoles into carnivorous, terrestrial, tetrapod adults. Continuous activity across this developmental transition is necessary to avoid predation and acquire resources. Like many other taxa, anurans show extensive plasticity in development and growth rates within and across life stages, rather than adhering to a physiological maximum [8–12]. However, in most anuran species, considerable growth occurs after metamorphosis [13,14] which may provide an opportunity to compensate for trait responses that would otherwise persist to negatively impact later-life stages [15,16]. Given this life history, anurans provide an excellent system to examine how trait plasticity varies across early development, as well as whether trait responses persist across life stages.

Given the advantages of anurans as a system, decades of studies have investigated developmental plasticity by rearing related individuals in different larval environments and measuring traits at metamorphosis. Across anuran lineages, this approach has revealed complex interactions between development speed and larval, metamorphic and post-metamorphic traits. Some studies provide evidence that the effects induced by larval conditions persist until metamorphosis [17–33], while others report pre-metamorphic compensation that reduces or eliminates the consequences of larval conditions on metamorphic traits [34–40]. Of the studies that have expanded sampling into the juvenile or adult stage, many have demonstrated that larger metamorphic size is correlated with larger post-metamorphic size and better post-metamorphic survival or performance [18,23,26,27,29,30,32,41–47]. However, some of these studies and others have shown that juveniles can fully or partially recover from a smaller metamorphic size [21,26,32,47–51]. These conflicting results are partially explained by the context-dependent relationship between development speed and metamorphic mass—the strength and direction of this relationship can vary based on the type, intensity and co-occurrence of environmental conditions and patterns of population-level local adaptation [11,52–56].

Since trait plasticity and its consequences can manifest in multiple forms within and across life stages, tracking traits across multiple timepoints is crucial to understand the mechanisms that underlie differences, or lack of differences, across ontogenetic shifts. Hundreds of studies over the past decades have assessed the effects of developmental conditions, including rearing density, temperature, salinity, pH levels, food availability and many more. However, most of these studies assess traits only at the metamorphic transition. Studies that sample at timepoints in addition to metamorphosis typically assess either only the larval period [19,20,29,37,38,40,57–68] or only the juvenile stage [21,32,41,45,47,49,69–71]. While these approaches have definitively demonstrated the scope and importance of early life plasticity for traits at metamorphosis, they have limited the field’s ability to draw conclusions about the consequences of trait plasticity across pre- and post-metamorphic timepoints. Notably, the few studies that have assessed traits within and across the larval and juvenile stages have been able to disentangle direct, indirect and interactive effects to show that the type, duration and timing of different larval conditions impacts compensation potential, or even over-compensation [23,30,36,50,51,55,56]. Taken together, these results not only demonstrate that the capacity for and effects of developmental phenotypic plasticity are complex even within the same taxonomic grouping, but also highlight the need to measure the same traits across multiple timepoints within and across life stages.

To address this knowledge gap, we assessed the effect of rearing density on larval duration, as well as morphology and survival during the larval period, at metamorphosis, and during the juvenile period up to eight months post-metamorphosis in wood frogs, *Rana sylvatica* (also referred to as *Lithobates sylvaticus* or *Boreorana sylvatica*). This species is responsive to interference competition (i.e. crowding) induced by population density [17,55–57,69]. We therefore predicted that the high-density rearing environment would produce smaller, later developing individuals, and were specifically interested in how these effects would be modulated across larval and juvenile life stages. Although natural environments comprise a combination of conditions that vary spatially and temporally and interact to alter the relationship between larval duration and metamorphic mass, we examined traits across a single type of condition known to have a strong effect on metamorphic traits—rearing density—to establish a baseline understanding of how trait responses vary *before* and *after* metamorphosis without confounding effects (e.g. food limitation). A more holistic understanding of phenotypic plasticity, compensatory ability and carry-over effects is essential, given increasing local environmental variability and accelerating global environmental change.

## 2. Methods

### 2.1. Egg acquisition and experimental set-up

We obtained six egg masses collected from Blackjack Pond, PA, USA (41°39′56.9982″ N, 80°30′43.9200″,W) and stored them to an indoor facility in Madison, WI, USA, at temperatures similar to those at the egg-collection site (4−5°C). After 2−12 days, the egg masses were shipped to Logan, UT, USA, and brought to ambient temperature in climate-controlled indoor facilities (humidity = 55%, set-point temperature = 24°C, 12 h : 12 h light : dark cycle). Although the set-point for the room’s air temperature was 24°C, the temperature of the water in tanks was slightly lower (21.1−23.8°C).

**Figure 1.**
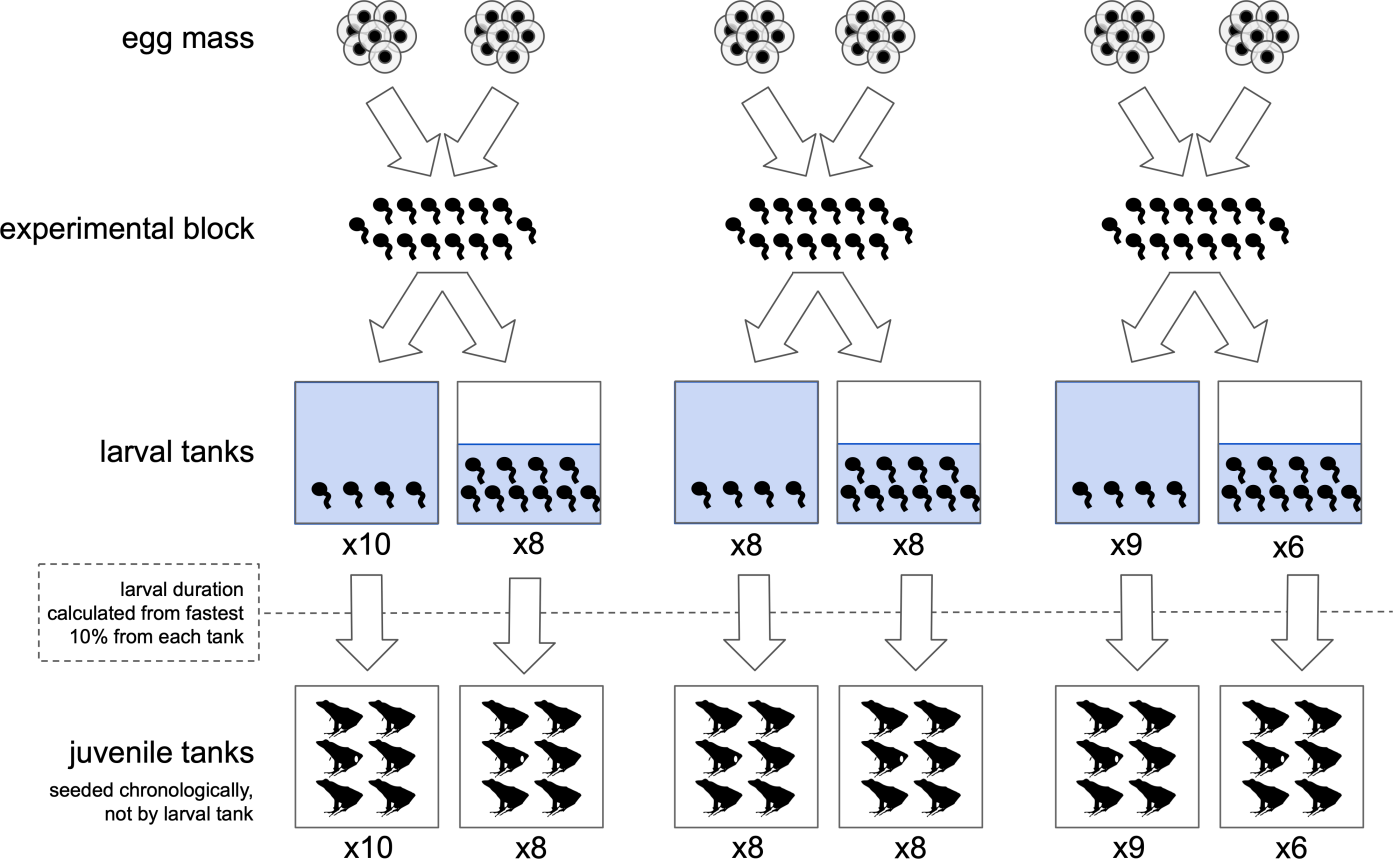
Experimental design for rearing individuals across life stages. Individuals from two egg masses were combined into each of three experimental blocks, which were then divided into low-density or high-density larval tanks. Low-density tanks had 40 individuals in 15.1 l (2.6 tadpoles l^−1^) and high-density tanks had 100 individuals in 7.6 l (13.2 tadpoles l^−1^). The number of larval tanks for each rearing density is listed under the tank icons. The first 10% of each tank to initiate metamorphosis (at least one forelimb emerged) were measured at metamorphosis completion. The first six individuals from each tank were also measured at metamorphosis and then seeded chronologically into juvenile tanks. The number of juvenile tanks for each rearing density is listed under the tank icons. Egg and larval silhouettes were made by the authors, and juvenile silhouettes are from PhyloPic.

When the hatched larvae (i.e. tadpoles) reached Gosner stage 23−26 [72], we created three experimental blocks, each comprising tadpoles from two of the six original egg masses (figure 1 and electronic supplementary material, figure S1). We combined egg masses because the number of viable individuals from each egg mass did not reach our targeted replication numbers. To retain genetic diversity (and thus, independence) across blocks, we paired two egg masses per block rather than sampling each block from a pool of all clutches, such that each egg mass was only represented in one block and all blocks came from different egg masses. This design retained higher relatedness within blocks and greater genetic diversity among blocks, which enabled us to control for block effects (i.e. effects of relatedness) in our analyses. We randomly selected and transferred tadpoles from each of these blocks into 5-gallon rectangular, opaque high-density polyethylene tanks (0.25 m length × 0.25 m width × 0.37 m height) filled with reverse-osmosis (RO) water to a density of 2.6 tadpoles l^−1^ (40 tadpoles in 15.1 l) for low rearing density and a density of 13.2 tadpoles l^−1^ (100 tadpoles in 7.6 l) for high rearing density. These densities are within the wide range reported from natural wood frog populations [73–75].

We used a randomized block design, but given variable hatching success and the number of experimental tanks required to ensure that repeated lethal sampling for other studies did not drop the density of tanks by more than 10%, the number of tanks varied for each rearing density and experimental block (figure 1 and electronic supplementary material, figure S1; high-density: A−8, B−8, C−6 tanks; low-density: A−10, B−8, C−9 tanks). We spatially aggregated blocks across two shelving units, but within each block, tanks differing in rearing density were alternated and distributed equally across each unit’s four vertical shelves to account for micro-climate differences.

### 2.2. Rearing details

We fed tadpoles daily ad libitum with boiled and pureed mixed greens such that neither density was food limited. We exchanged half the water volume for fresh RO water once per week and removed faecal matter with a net twice per week to maintain water quality. To maintain starting densities—our experimental variable of interest—we adjusted water volumes weekly following water changes to account for tadpole death and lethal sampling for other studies.

We visually checked tadpoles once daily for general health and signs of forelimb emergence. We did not remove dead tadpoles, since they can provide visual or chemical cues that conspecifics use to assess habitat quality and adjust developmental or growth rate. When at least one forelimb had emerged from an individual, we removed that individual from the rearing tank and placed it in a plastic container (7.0 × 10.5 × 5.7 cm) tilted to create a shallow pool of RO water, with pebbles and sphagnum moss to prevent drowning. We checked each metamorphosing tadpole once daily for metamorphosis completion (i.e. tail resorption: tail length less than 1 mm).

Upon tail resorption, metamorphs were transferred into a plastic tank (30.8 × 20.5 × 15.1 cm) equipped with RO water, moist coconut fibre and sphagnum moss. We distributed metamorphs across tanks (maximum 12 individuals per tank) chronologically by timing of forelimb emergence, experimental block and rearing density. This approach resulted in low within-tank variation in post-metamorphic age (tank mean ± s.d. age range = 5 ± 2.3 days, minimum age range = 2 days, maximum age range = 10 days). We checked tanks daily for general health and level of the RO water source, misted the tanks with RO water every 1−3 days and fed the juveniles either fruit flies or cricket nymphs ad libitum every 2−4 days.

We limited our sampling for metamorphic and post-metamorphic traits to the earliest developing individuals, which ensured experimental feasibility given the long and extremely variable developmental time within and between density treatments. Thus, only the first six individuals from each larval tank that initiated metamorphosis were retained for juvenile tanks. We terminated the larval portion of the experiment for each experimental block once the first six individuals from every tank of that block had at least one forelimb emerged. In addition to ensuring logistical feasibility, this sampling approach is ecologically realistic as the percentage of each tank population that we retained for juvenile tanks (high density: 6%, 6/100; low density: 15%, 6/40) is within the range of cohort metamorphosis percentage in wild populations of wood frogs (3–8% [74]). This sampling design provides a conservative estimate of the consequences of rearing density and focuses the scope of our conclusions of metamorphic and post-metamorphic traits to the earliest developing individuals from each tank.

### 2.3. Developmental data collection

To assess differences in larval duration and metamorphosis duration, we calculated the percentage of tadpoles that had initiated metamorphosis for each week and larval tank. We continued to record the larval duration of all individuals with forelimb emergence until experimental block closure so that we could assess the proportion of each tank metamorphosed at the end of the larval portion of the experiment. Given the variation in developmental timing across tanks within each block, we recorded larval duration for up to 20–25% of each high- and low-density tank, respectively. However, to ensure that we compared developmental speed between rearing densities for the same *proportion* of each tank, we calculated larval duration based on the first 10% of individuals to metamorphose for all but three tanks, which equates to 294 total individuals (figure 1 and electronic supplementary material, figure S1). Upon block closure, we manually counted all surviving tadpoles and then euthanized them in MS-222 and stored them in 10% neutral-buffered formalin. Within 4−6 months of fixation, we staged tadpoles and used the average of minimum and maximum stages if tadpoles appeared to be in transition between stages. We calculated a tank-level mean developmental stage, taking into account any metamorphosed individuals that had previously been removed from the tanks by assigning them as Gosner stage (GS) 42.

We did not mark individual metamorphs after tail resorption (hereafter, juveniles), so for juvenile tanks we calculated a tank-level mean larval duration from the larval durations of each tank’s seeded individuals.

### 2.4. Morphometrics data collection

We used digital photography to measure larval morphology at 5−6 timepoints, beginning at two weeks after tank seeding and ending with block closure at 9−10 weeks. Within each block and sampling point, we pseudo-randomly selected a set of tanks for each rearing density (*n* = 5), such that we sampled across the entire variation of the block at 2−3 timepoints. Some tanks were sampled in back-to-back timepoints, and some tanks were sampled every 2−3 timepoints. During each larval sampling point, we photographed 10 tadpoles dorsally with a digital single-lens reflex camera (EOS Rebel T3, Canon, Tokyo, Japan) equipped with a macro lens (EF-S 18-55mm f/4-5.6 IS STM; Canon). We used ImageJ to measure total length, body length, tail length, head width, body width and tail-muscle width [76].

To assess carry-over effects of the larval density on metamorphic and post-metamorphic size and body condition, we used a digital scale (Analytical Balance LA204E, Mettler Toledo, Greifensee, Switzerland) and digital calipers (AOS Absolute Digimatic, Mitutoyo, Takatsu-ku, Kawasaki, Kanagawa, Japan) to measure morphology at tail resorption for the first six individuals to metamorphose from each tank and at six post-metamorphic sampling points (1–2, 4–6, 8–10, 12–14, 16–18 and 30−31 weeks). We gently blotted each froglet with a Kimwipe then measured the damp mass to 0.0001 g and body length (i.e. snout–vent length), right forelimb, right thigh and right tibia to 0.01 mm [77].

Larger mass in an individual can result from larger skeletal size or more stored energy reserves (i.e. fat, protein, lean mass), so we calculated a metric of body condition—the scaled mass index (SMI)—for each individual at each timepoint using the following equation [78]:


$${{\text{SMI}}_{\text{individual}}={\text{Mass}}_{\text{individual}}\left(\frac{{\text{SVL}}_{\text{population}}}{{\text{SVL}}_{\text{individual}}}\right)}^{\text{bSMA}},\;\text{bSMA}\;=\;\frac{\text{bOLS}}{r}.$$

In this equation, bOLS represents the slope from the ordinary least squares (OLS) regression of mass on body length, and *r* represents the Pearson’s correlation coefficient between mass and snout–vent length (SVL), both calculated using log-transformed variables. We calculated SVL using the metamorphic and post-metamorphic data from all individuals retained for the juvenile tanks. Calculating SMI in this way has been validated in ranids as an accurate assessment of energy stores [79].

Since we did not track individual identities of juveniles after metamorphosis, we calculated a tank-level mean metamorphic mass and mean metamorphic body condition based on each tank’s seeded individuals.

### 2.5. Survivorship data collection

We determined larval survivorship weekly using ImageJ to manually count individuals in one photo per tank [80]. We determined juvenile survivorship weekly for each tank by manually counting individuals. We calculated the tank-level survivorship proportion for each larval and juvenile tank by dividing the number of live individuals by the tank’s seeded number, adding the number of individuals that had metamorphosed to the number of live individuals for larval tanks. As part of related experiments, we lethally sampled 1−2 individuals from each sampled tank at each sampling point, and we subtracted this number from the seeded number during survivorship calculations.

### 2.6. Statistical analyses

We used generalized linear mixed models and linear mixed models using the ‘lme4’ package in R and RStudio [81–83] to assess additive and interactive effects on numeric morphological metrics (larval duration, developmental stage and juvenile body condition) and binary status (metamorphosis and survivorship). We controlled for non-independence between tanks from the same experimental block by including a random effect of tank nested within block or a random effect of block in our statistical analyses. For all response variables, we constructed a set of candidate models and used the *anova* function in the ‘stats’ package [82] and the *AICc* function in the ‘MuMIn’ package [84] to compare nested and non-nested candidate models, respectively.

We checked model assumptions using the diagnostic plots and functions in the ‘DHARMa’ package [85] and explored transformations of the response variables using the natural logarithmic function when diagnostic plots indicated deviations from normality. The details for candidate models and the best-fit model from each hypothesis test, including the model structure and transformations, are provided in the electronic supplementary material (tables S1–S9).

## 3. Results

### 3.1. High-density rearing environment delays development

**Figure 2.**
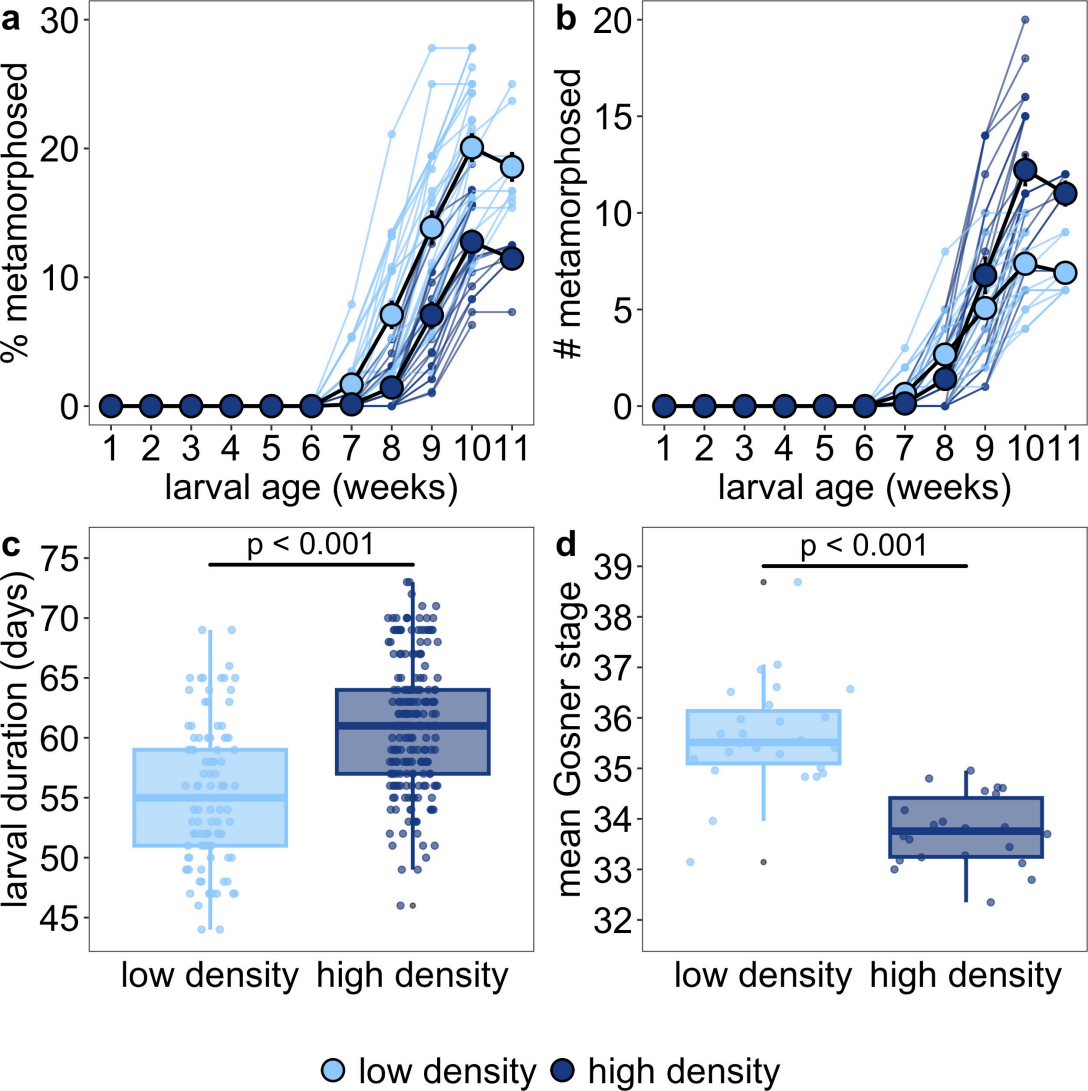
High density delayed development when considering (a) the percentage of seeded individuals initiating metamorphosis each week, (b) the raw number of individuals initiating metamorphosis each week, (c) larval duration across experimental blocks for the first 10% of individuals from each tank to initiate metamorphosis and (d) tank-level mean developmental stage across blocks. For panels (a,b), tanks are plotted separately as coloured points for each week and connected across weeks as coloured lines. Means ± 1 s.e.m. are plotted by rearing density in solid black lines. Note that the percent and raw number metamorphosed decrease in week 11 because the calculations in week 11 are from only one block that finished the experiment one week later than the other two blocks. For panel (c), individuals are plotted separately as coloured points. For panel (d), tank means are plotted separately as coloured points with outliers in grey.

The earliest metamorphosis was initiated within a low-density tank at 44 days, and individuals in high-density tanks started metamorphosing 2 days later. By the end of the experiment, more individuals from high-density tanks had metamorphosed; however, the percentage of individuals that had metamorphosed was 7.8% lower in the high-density treatment (mean 13.4 ± s.d. 3.4% [range 6.3−20.8%] compared with the low-density treatment (21.2 ± s.d. 4.3% [range 15.4−27.8%]) (figure 2a,b and electronic supplementary material, table S1a). The delaying effect of high density on development was consistent whether calculating the percentage of metamorphs relative to the *seeded* number of individuals or relative to the *surviving* individuals (electronic supplementary material, table S1a,b).

The high-density rearing environment delayed metamorphosis by an average of 6 days compared with the low-density rearing environment (figure 2c and electronic supplementary material, table S2). By the end of the experiment, both low-density and high-density tanks had non-metamorphosing individuals that had barely advanced in development since seeding (GS 26) and individuals that were close to forelimb emergence (GS 41). However, the tank-level mean developmental stage was about two stages earlier for high-density tanks compared with low-density tanks (figure 2d and electronic supplementary material, table S3). This developmental difference between mean stages equated to stage 34 (i.e. developing a fifth digit on the hindlimbs) and stage 36 (i.e. separation of first three digits on the hindlimbs).

### 3.2. High-density rearing environment depresses early larval growth but compensatory growth eliminates size differences in later larval development

**Figure 3.**
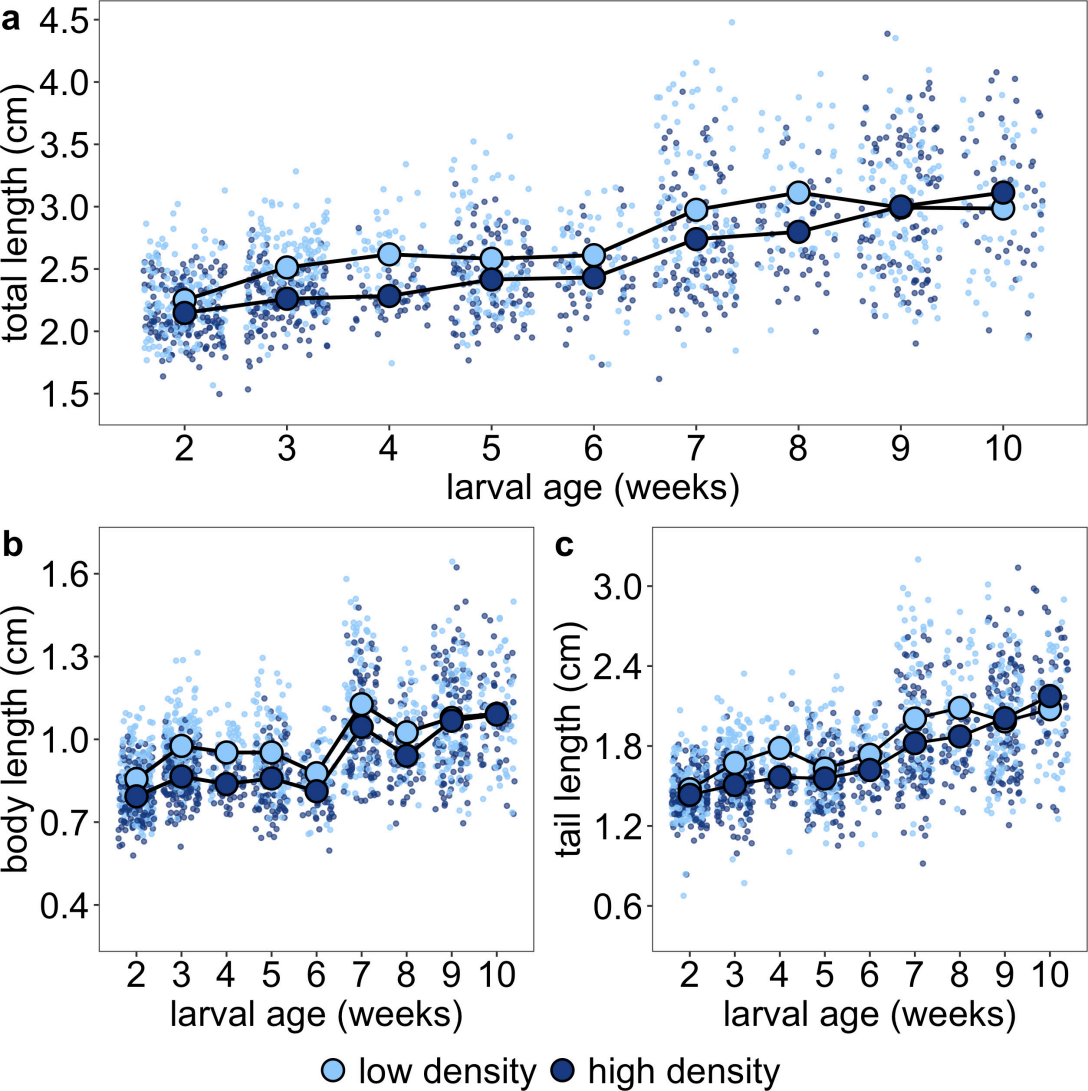
High density decreased early larval size but compensatory growth in high-density tanks resulted in similar-sized tadpoles by the close-out of larval tanks when considering (a) total length, (b) body length and (c) tail length, all measured in centimetres. Each small point represents an individual at each sampling point, and each large point outlined in black represents a time series of mean ± 1 s.e.m. across all sampling points, plotted separately by rearing density.

High-density tadpoles were smaller than low-density tadpoles within two weeks after the start of the experiment, regardless of whether we considered total length or body length as the size metric (figure 3a,b and electronic supplementary material, table S4a,b). Although the size difference decreased in weeks 5−7, it did not disappear until weeks 9−10 when individuals from high-density and low-density tanks had similar total, body and tail lengths (figure 3). Despite having shorter tails until weeks 9−10, tadpoles from high-density tanks had similar *relative* tail length (to total length and body length) across the larval period compared with tadpoles from low-density tanks, suggesting that tadpoles from both densities had similar morphologies despite size differences. Body width, head width and tail muscle width showed similar patterns to those for length measurements in that they were smaller for high-density tadpoles until weeks 9−10 (electronic supplementary material, table S4).

### 3.3. Later metamorphosing individuals, regardless of rearing density, are smaller but in slightly better body condition

**Figure 4.**
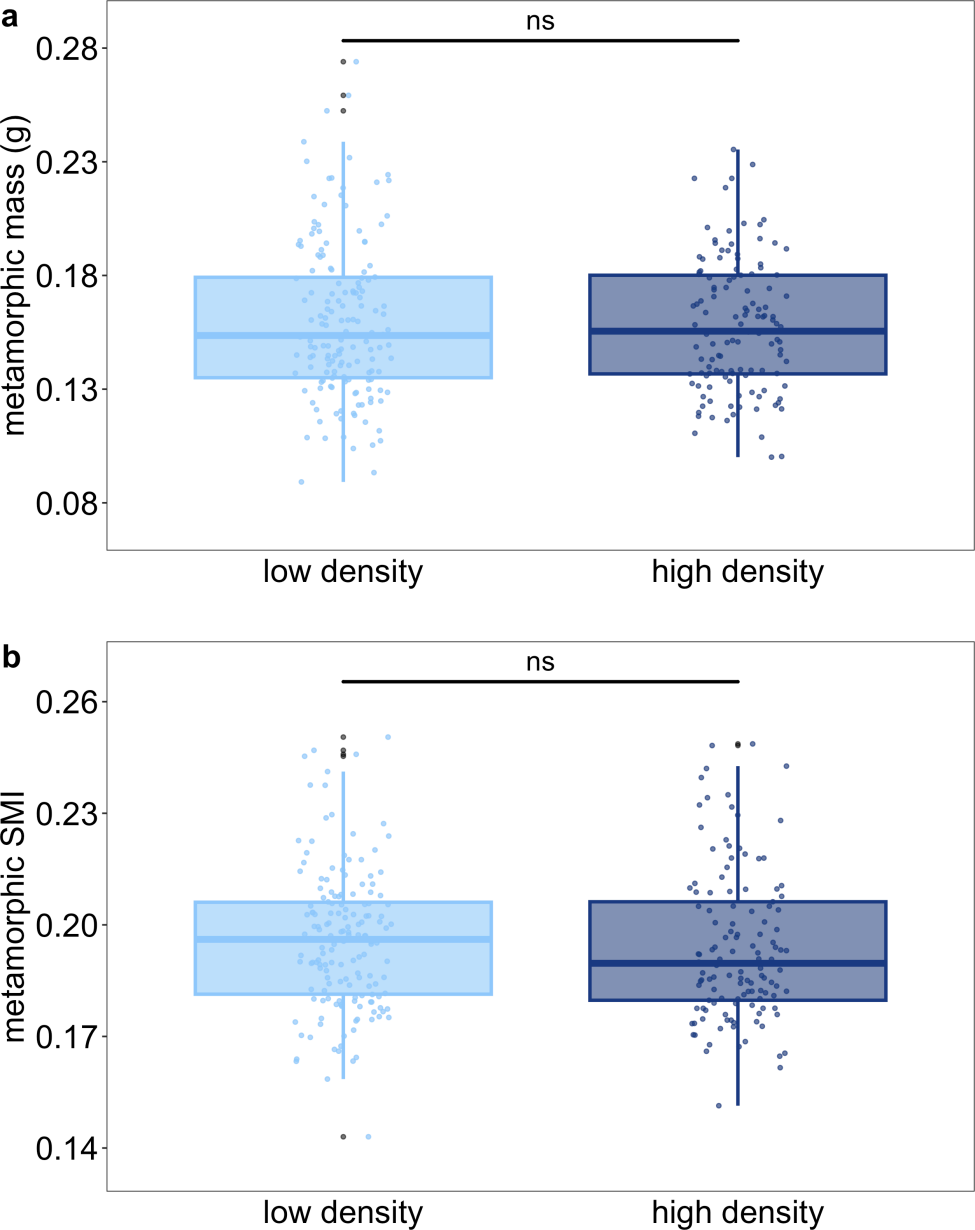
Rearing density had no significant effect on metamorphic mass (a) or SMI (b) for the six fastest developers in each tank. Each point represents an individual with outliers in grey.

**Figure 5.**
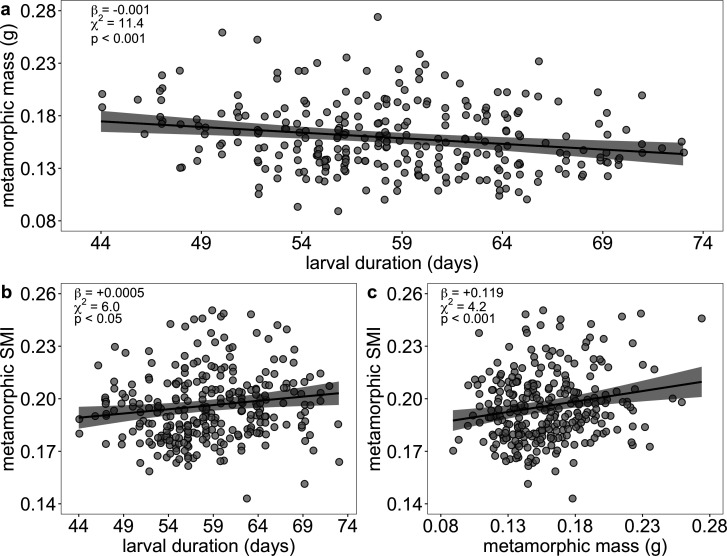
Larval duration was negatively correlated with mass at metamorphosis (a) and positively correlated with SMI at metamorphosis (b), while mass at metamorphosis positively correlated with SMI at metamorphosis (c). Each point represents an individual. For each panel, the solid black line indicates predicted values ±95% confidence intervals from the best-fit generalized linear mixed model fitted with Gaussian errors, for which the coefficient estimate (*β*), *X*^2^ value and *p*-value are listed in the upper-left corner.

Given the logistical complications of tracking traits across life stages for a species in which developmental speed varies widely, we limited data collection for metamorphic morphology and post-metamorphic data to the earliest six individuals that initiated and completed metamorphosis from each tank (total individuals: low density—158, high density—127; figure 1 and electronic supplementary material, figure S1). Among these individuals tracked through the juvenile stage, larval duration varied by 29 days (mean 58.4 ± s.d. 6.1 days [range 44−73 days]), but rearing density was not a significant driver of this variation (electronic supplementary material, table S5). At metamorphosis completion, mass varied threefold (mean 0.159 ± s.d. 0.032 g [0.089−0.274 g]), but rearing density did not affect mass among individuals (figure 4a). Instead, larval duration was the most influential factor in observed variation in mass (figure 5a and electronic supplementary material, table S6a). In terms of length metrics, rearing density only showed an effect on forearm length—individuals from high-density tanks had slightly shorter forearms (electronic supplementary material, table S6c–f). Although we reduced the water level as needed to maintain relative densities in some low-density tanks, these reductions did not influence variation in metamorphic mass or the relationship between metamorphic mass and larval duration (electronic supplementary materials, figure S2 and table S7).

Although correlated with metamorphic size, body condition was considered as a separate metric to assess whether differences in size reflected differences in underlying health among individuals retained for juvenile tanks. Relative to body mass, body condition at metamorphosis showed less variation (mean 0.186 ± s.d. 0.019 [range 0.134−0.241]), and rearing density did not affect variation in body condition among individuals (figure 4b and electronic supplementary material, table S6b). Instead, increased mass and, to a lesser extent, larval duration significantly improved body condition (figure 5b,c and electronic supplementary material, table S6b). Notably, the effect of larval duration on body condition was weaker and in the opposite direction of its effect on metamorphic mass—although later developers were smaller at metamorphosis, they had a slightly improved body condition relative to earlier developers.

### 3.4. No evidence for compensatory increase in growth or body condition after metamorphosis

**Figure 6.**
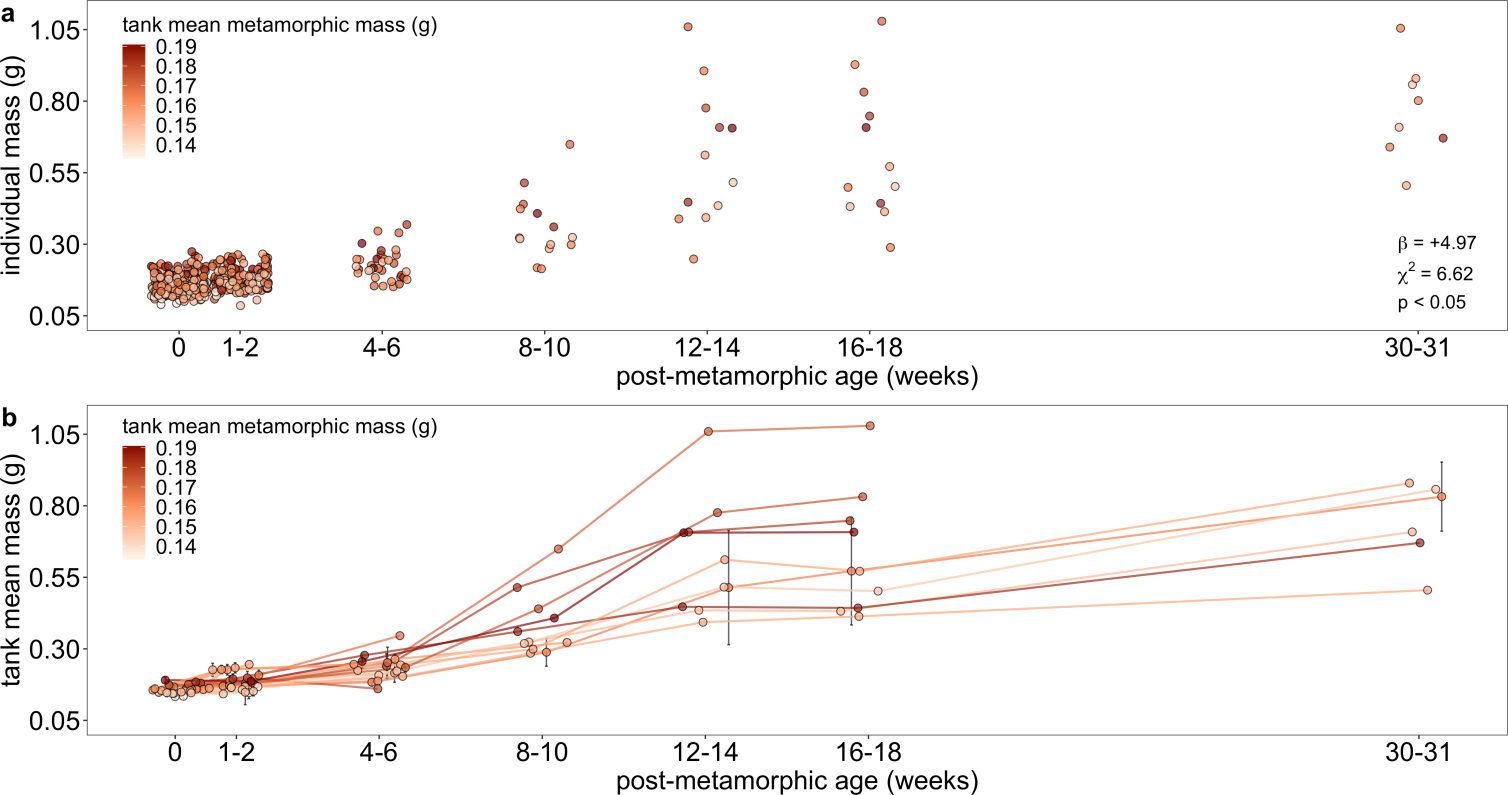
Tank-level metamorphic mass influenced post-metamorphic mass of individuals (a) and mean mass of tanks (b) up to 30−31 weeks after metamorphosis. (a) Each point represents an individual’s mass at metamorphosis and six sampling points after metamorphosis with tank-level mean metamorphic mass signified across a colour gradient of smallest (light tan) to largest tanks (dark red). The coefficient estimate (*β*), *X*^2^ value and *p*-value for tank mean metamorphic mass are listed in the lower-right corner from the best-fit generalized linear mixed model fitted with Gaussian errors. (b) Each point connected by a line represents tank-level mean mass at metamorphosis and six sampling points after metamorphosis with tank-level mean metamorphic mass signified across a colour gradient of smallest (light tan) to largest tanks (dark red). Tanks comprising larger metamorphs (darker coloured points and corresponding lines) tended to persist longer and grow larger than tanks comprising smaller metamorphs (lighter coloured points and lines). Means ± 1 s.e.m. are plotted when available, but tank means shown without error bars indicate a single surviving individual for that tank and sampling point.

Individuals in tanks comprising larger metamorphs tended to maintain a larger size after metamorphosis than individuals in tanks comprising smaller metamorphs, regardless of rearing density (figure 6 and electronic supplementary material, table S8a). This pattern was consistent across most other morphological length metrics (i.e. snout–vent, thigh and tibia; electronic supplementary material, table S8c–f). Forearm length was the one exception, since only post-metamorphic age, but not metamorphic mass, positively influenced post-metamorphic forearm length (electronic supplementary material, table S8d). Unlike body size, body condition (mean SMI) varied inconsistently across tanks after metamorphosis, but the general trend comprised a slight decrease in body condition with post-metamorphic age (electronic supplementary material, table S8b).

### 3.5. High density decreases larval survivorship and smaller metamorphic size predicts lower post-metamorphic survivorship regardless of rearing density

**Figure 7.**
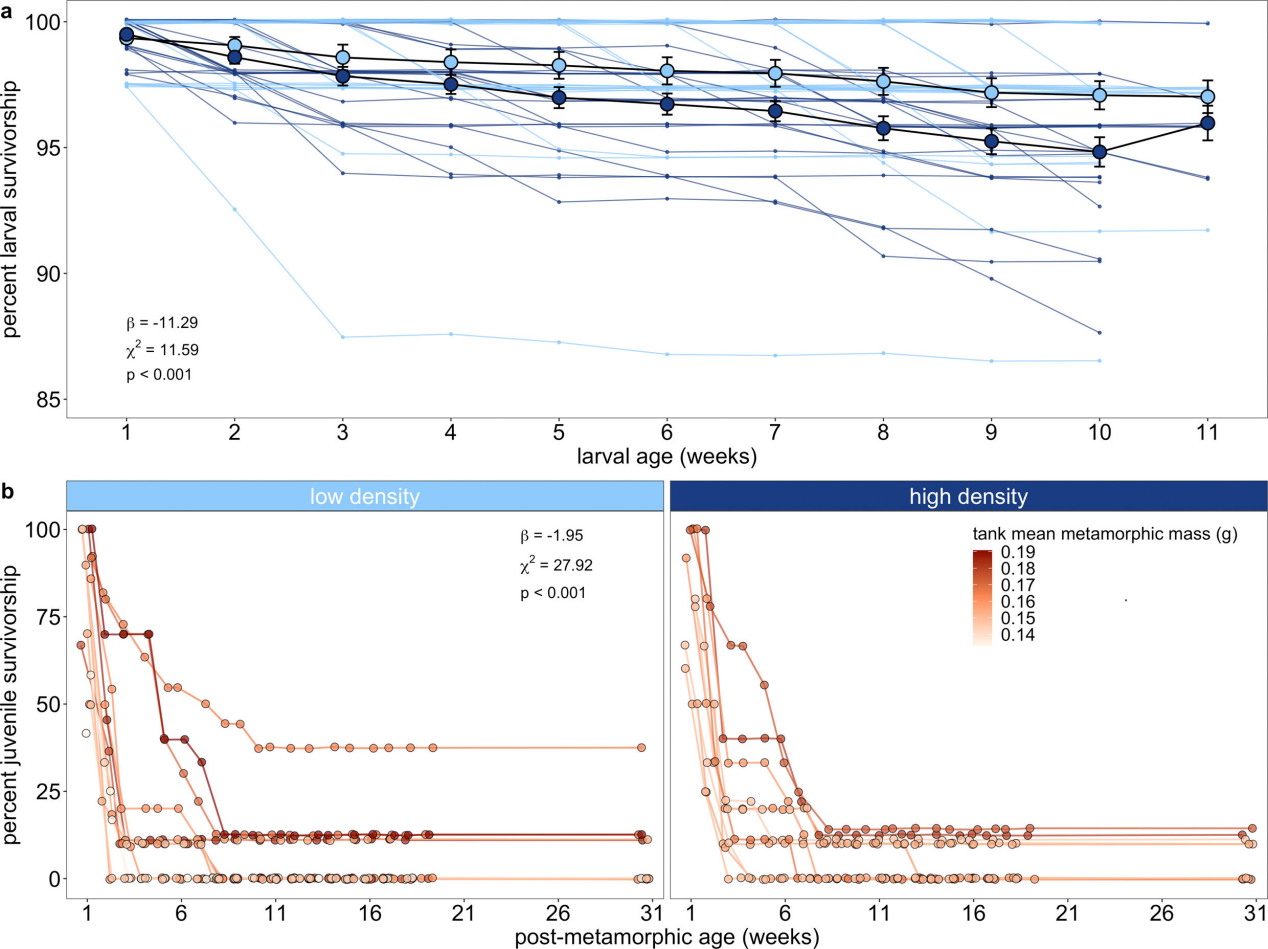
Weekly survivorship during the larval period (a) and up to 30−31 weeks after metamorphosis (b). (a) Each small coloured point represents the percent of seeded individuals alive at each sampling point for each tank, with rearing density indicated by colour, and each large coloured point outlined in black represents the rearing-density level means of percent survivorship. Note that survivorship increases in week 11 because the calculations are from one experimental block that finished the experiment one week later. The coefficient estimate (*β*), *X*^2^ value and *p*-value for the interaction between rearing density (reference = high density) and age are listed in the lower-left corner for the best-fit generalized linear mixed model fitted with binomial errors. (b) Each connected point represents the percent of seeded individuals still alive at each sampling point for each tank, plotted separately in panels by larval rearing density. Lines connecting points indicate a time series for a tank. Tank-level mean metamorphic mass is signified across a colour gradient of smallest (light tan) to largest (dark red) metamorphs, which is the same for both panel plots. The coefficient estimate (*β*), *X*^2^ value and *p*-value for the interaction between rearing density (reference = high density), tank-level mean metamorphic mass, larval duration and post-metamorphic age, which are the same for both panel plots, are listed in the upper-right corner of the left panel for the best-fit generalized linear mixed model fitted with binomial errors.

Survivorship was high (greater than 85%) throughout the larval period and to the close-out of larval tanks, but survivorship slightly decreased for both rearing densities with larval age. High-density tanks had lower larval survivorship, on average, than low-density tanks throughout the larval period (figure 7a and electronic supplementary material, table S9a).

Juvenile survivorship dropped rapidly during the first two weeks after metamorphosis, then slowed around three weeks before stabilizing at a low level around eight weeks that persisted up to eight months post-metamorphosis (figure 7b). A three-way interaction between rearing density, mean metamorphic mass and mean larval duration influenced survivorship after metamorphosis (electronic supplementary material, table S9b). For juvenile tanks whose individuals were reared at low density, lower survivorship was primarily driven by smaller mean metamorphic mass. However, juvenile tanks whose individuals were reared at high density showed a negative effect of smaller mean metamorphic size on survivorship only if they comprised early developing individuals.

## 4. Discussion

Our results demonstrate that phenotypic response to the larval environment has different effects across life stages for fitness-related traits. Although wood frogs are well-represented in studies of density effects on development, our study is one of the few to report trait values for metamorphosis and another life stage [57,59,61,69]. As expected, high rearing density reduced early larval growth rate, decreased larval survival and extended larval duration. However, our study’s repeated sampling during the larval stage enabled us to identify that larval growth-rate plasticity compensated for the early suppressive effects of rearing density by mid-to-late larval development. Variation in larval duration, rather than rearing density, influenced metamorphic mass for the earliest developing individuals. Repeated sampling during the juvenile stage demonstrated the lasting impact of larval duration and metamorphic mass on juvenile growth and survivorship—unlike in the larval stage, juveniles did not compensate for a smaller metamorphic body size. Our study adds to the growing literature that examines trait responses to rearing density within or across life stages in other ranids [21,58,70,71,86] or in non-ranids [22,30,31,40,45,46,60,87] and documents the value of expanding sampling within and across stages to track the mechanisms and consequences of shifts in mean trait values, which will be essential for predicting population and species persistence in the face of rapid global environmental change.

### 4.1. Growth-rate plasticity during larval period compensates for early development effects of rearing density by metamorphosis

As we predicted and consistent with other studies in wood frogs, the high-density rearing environment reduced early larval growth rate [57,87,88]. However, the compensation of high-density tadpoles was an unexpected result—growth rate increased in high-density tanks relative to low-density tanks, such that rearing density had no effect on morphological differences by mid-to-late larval development and metamorphosis. Crowding has been shown to decrease larval growth in wood frogs, but these effects persisted close to metamorphosis [87,88] or were minimized depending on food levels [57,59]. We speculate that the growth suppression and compensatory increase in larval growth rate observed for high-density tanks were modulated by physiological and/or behavioural mechanisms, given that they occurred within a constant environment and ad libitum food resources, similar to recent work in bufonids [40]. Although other ranid studies have demonstrated compensatory growth during the larval period, these responses were initiated upon the removal of a non-density stressor in the larval stage [19,36]. Our results emphasize the importance of assessing phenotype throughout the larval stage, since this approach allowed us to determine that the lack of morphological variation between rearing densities in later larval weeks was due to compensatory growth-rate plasticity, rather than insensitivity to the rearing environment.

Our study’s rearing conditions were sufficient for species-typical growth and development rates, since the ranges for larval duration and metamorphic mass fall within previously reported ranges for wood frogs [17,59,88,89], and survivorship was higher or comparable to other studies [55,56,69,88]. Early growth suppression due to interference competition, rather than limiting food resources, may have been responsible for the lower weekly survivorship and slower larval development in high-density tanks. Previous studies have suggested that metamorphosis in anurans is contingent on tadpoles reaching a minimum size [15,90,91]. If this is the case, individuals in our study reared at high density may have needed a longer time to reach a body size and/or body condition sufficient for metamorphosis, given the early growth suppression. We speculate that the ability to compensate for detrimental early life conditions may be adaptive in wood frogs, given their wide geographical distribution and extreme variation across populations in densities experienced during development.

### 4.2. Metamorphosis does not decouple effects of larval duration from juvenile stages—lasting advantage of earlier development and larger size at metamorphosis

Altogether, our results suggest that while some individuals (the earliest developers) can compensate for the negative effects of high larval density on life-history traits, trait values at metamorphosis have lasting effects on juvenile phenotype. Individuals tracked from metamorphosis through juvenile development showed substantial variation in larval duration (29 days) and metamorphic mass (threefold) that was not attributable to rearing density. Individuals that metamorphosed on the earlier end of the 29-day range were larger, and metamorphic mass strongly influenced juvenile mass up to at least eight months after metamorphosis. The persistent effect of metamorphic size in the juvenile stage was not due to variation in body condition or fixed growth rates. Growth rates varied, but in a similar way across early to late developers and metamorphic sizes—growth was slow initially then increased after four−six weeks and again after 16−18 weeks. Although the effects of metamorphic size were apparent within the first month after metamorphosis—a time period in which most studies conclude their sampling—the majority of growth occurred in later weeks. By continuing to collect data at later timepoints in a controlled high-resource environment, our study confirms that metamorphic mass matters across periods of both low and high growth rates during the juvenile stage.

Our results more than triple the known time frame for effects of the metamorphic state to persist in the juvenile stage for an experimental study that assesses interference competition in wood frogs. The only other experimental study to track juvenile size after metamorphosis in wood frogs reared at different densities found a similar pattern between larval period and metamorphic size—early developers tended to be larger at metamorphosis and maintained a size advantage up to nine weeks after metamorphosis [69].

The effects of metamorphic size can extend beyond the juvenile stage to influence lifetime fitness in ranids. Multiple observational studies of wild populations have determined that larger metamorphic size is associated with larger adult size [92], which increases reproductive opportunities [92–94] and reproductive success [74,92,94,95]. Although in most anurans a substantial portion of adult size is attained through post-metamorphic growth, the capacity to increase larval growth may be especially impactful for ranids, which tend to metamorphose at a larger size relative to their adult size and have a larval period that comprises a larger proportion of time to maturity compared with other anurans [14,15]. In wood frogs, age at first reproduction can be as short as 1 year [92], but since juveniles must survive overwintering before the annual spring breeding occurs, a larger metamorphic size could impact survival to and success in the first breeding event. Thus, for wood frogs, size gained in the larval stage that carries over to a larger metamorphic size may significantly and quickly pay off in the juvenile and adult stages, especially if opportunities to increase growth rate are limited in the terrestrial environment. More studies are needed that track juveniles up to and beyond the age of first breeding to assess the carry-over effects of larval and/or metamorphic traits.

### 4.3. Being bigger is mostly (but not always) better—the effect of metamorphic mass on juvenile survivorship depends on rearing density

Our results of low juvenile survivorship, especially in the first few weeks after metamorphosis, are consistent with field studies in wood frogs and other ranids. However, our study’s approach of tracking known juveniles in controlled environments enables us to more confidently identify the intrinsic mechanisms driving observed patterns by removing the variation in habitat and environmental conditions inherent to field studies. Larger body size in the terrestrial juvenile stage had clear and early fitness advantages in our study. First, the fastest decrease in survivorship across all tanks coincided with low growth during the first few post-metamorphic weeks, before levelling off as growth increased. Second, survivorship of tanks with smaller individuals decreased much more quickly than tanks with larger individuals and was significant even though calculating tank-level means inherently reduced the data’s resolution (i.e. condensed threefold individual-level variation into 0.75-fold group-level variation).

Since all individuals experienced similar post-metamorphic environments in our study, variation in juvenile survival was not attributable to extrinsic factors, such as predation, access to water, food resources or size-dependent interactions among these factors. Instead, early juvenile survivorship depended on body size and/or some other intrinsic factors that may or may not vary as a function of body size in anurans, such as the ability to catch, consume and process prey [45], fat stores [96], aerobic capacity [97], activity level [30] or a combination of these factors. In support of this, we found that the relationship between metamorphic mass and post-metamorphic survival was more complex in tanks with individuals reared at high density. Of these tanks, those with smaller individuals experienced lower survivorship only if they contained *earlier developing* individuals. This suggests that the degree of larval compensation required to overcome growth suppression can induce trade-offs with juvenile survival. For example, rapid acceleration of growth in a tadpole—while enabling that tadpole to more quickly reach the smaller range of body size sufficient for metamorphosis—may limit the development of organ systems, acquisition of energy stores or learning of behaviours beneficial for post-metamorphic survival. Increasing evidence points to developmental trade-offs emerging as ‘hidden’ traits, such as stress response [51], immune function [41,98], gonadal maturation [99], behavioural syndromes [100] and brain morphology [101–103]. As noted in a recent review [104], future work should investigate a range of traits within the same organism to build a mechanistic understanding of how phenotypic plasticity manifests to influence survival and whole-organism performance metrics across life stages.

## 5. Caveats and considerations

A strength of our study design is sampling at multiple timepoints within and across life stages to gain a fine-scale resolution of developmental environment effects. However, this strength also posed a logistical constraint given the wide variation in developmental time in this species. Consistency across different metrics in our study suggests that patterns at metamorphosis were not simply due to our sampling design of measuring traits in the earliest developers, which represent a small overall proportion of the experimental population. First, the similarity in metamorphic mass between rearing densities is a pattern consistent with the similarity in late larval stage size metrics between rearing densities (which we sampled randomly). Second, the 6 day difference in larval duration between rearing densities is consistent with the two-stage difference in the developmental stage of all remaining tadpoles at the close-out of tanks. The duration of transitioning across these two Gosner stages corresponds to a difference of less than a week in wood frogs (SM Strobel, personal observation) and other species [105–109]. Moreover, the difference of 6 days and two Gosner stages measured in our study is within the range of those reported in other studies for wood frogs [57,59,69,87,89] and other ranids [86,110,111] that sampled a great proportion of the experimental population. This similarity demonstrates that the small-magnitude developmental delay induced by high rearing density in our study is reproducible and ecologically relevant, rather than an artefact of sampling the earliest developers. Third, although we only retained the earliest six developers from each tank for metamorphic and juvenile measurements, we preserved and stored (in 10% neutral-buffered formalin) all individuals that metamorphosed until block closure. We measured SVL (mm) in these fixed individuals—which represent 15% and 25% of high- and low-density tanks, respectively—and found a similar result to the patterns observed with sampling 6% and 15% of high- and low-density tanks, respectively (electronic supplementary materials, figures S3 and S4). These lines of evidence suggest that measuring metamorphic traits for more individuals would not have changed our observed pattern that larval duration, rather than rearing density, influenced size at metamorphosis.

Although we cannot exclude the possibility that sampling all individuals at metamorphosis and retaining all individuals for juvenile tanks would have produced different results, we emphasize that, in wild cohorts, the percentage of wood frog tadpoles that metamorphose is within the range (3–8%) of what we sampled for this study [74]. Thus, our observation that some individuals can compensate for the early negative impacts of high rearing density on growth has ecological relevance for considering how developmental environment can impact population persistence and, thus, selection on growth-rate plasticity. Tracking the growth and survival of individuals who metamorphose—even if it comprises a small proportion of the cohort, as has been documented for wild populations—is critical to understand the factors that influence survival to reproductive age, and ultimately selection on heritable traits associated with plasticity. Other field and laboratory studies in ranids that measure post-metamorphic traits also sample a small proportion of the initial population [32,74]. Rather than invalidating our observed patterns at metamorphosis, our sampling design merely constrains our interpretations of the effect of rearing density and developmental speed on metamorphic size to the 29 day range represented by the earliest developers, as has been noted in other developmental plasticity studies in anurans [32,101].

## 6. Conclusions

Early development shapes the trajectory of individuals, and the degree to which larval trait responses persist in later life stages ultimately influences the phenotypic variation upon which selection acts. However, only a few studies on anuran developmental plasticity track traits across both the larval and juvenile stages, which has limited the field’s ability to account for variation in sensitivity across developmental stages when predicting trait responses. In our study, we found that high rearing density depressed early larval growth in wood frogs, but this effect was countered by increased growth rate in later larval stages. For the earliest developing individuals, metamorphic mass was inversely related to larval duration, and larger metamorphs grew into larger juveniles. A larger metamorphic mass improved juvenile survivorship except for individuals reared at high density—for these individuals, smaller metamorphs survived as well as larger metamorphs if they experienced a longer larval duration. Our study’s approach to measure trait responses across multiple timepoints in larval development and in the juvenile stage improves our ability to disentangle the mechanisms underlying the capacity for and consequences of phenotypic plasticity in a well-studied vertebrate system.

## Data Availability

The datasets and code supporting this article have been uploaded as part of the electronic supplementary material [112].
